# Overexpression of a *Zea mays* Brassinosteroid-Signaling Kinase Gene *ZmBSK1* Confers Salt Stress Tolerance in Maize

**DOI:** 10.3389/fpls.2022.894710

**Published:** 2022-05-06

**Authors:** Lei Liu, Yanchao Sun, Pengcheng Di, Yakun Cui, Qingchang Meng, Xiaming Wu, Yanping Chen, Jianhua Yuan

**Affiliations:** ^1^Provincial Key Laboratory of Agrobiology, Institute of Food Crops, Jiangsu Academy of Agricultural Sciences, Nanjing, China; ^2^College of Life Sciences, Nanjing Agricultural University, Nanjing, China

**Keywords:** antioxidant defense enzyme, maize, protein interaction, reactive oxygen species, salt tolerance, ZmBSK1

## Abstract

Salinity has become a crucial environmental factor seriously restricting maize (*Zea mays* L.) growth, development and productivity. However, how plants respond to salt stress is still poorly understood. In this study, we report that a maize brassinosteroid-signaling kinase gene *ZmBSK1* plays a significant role in salt stress response. Expression pattern analysis revealed that the transcript level of *ZmBSK1* was upregulated by NaCl treatment both in maize leaves, roots, and stems. Phenotypic and physiological analysis showed that overexpression of *ZmBSK1* in maize improved salt tolerance by reducing the malondialdehyde (MDA) content, the percentage of electrolyte leakage, O_2_^−^ and H_2_O_2_ accumulation under salt stress, relying on the increases of antioxidant defense enzyme activities and proline content. qRT-PCR analysis showed that overexpression of *ZmBSK1* also positively modulated the expression levels of reactive oxygen species (ROS)-scavenging and proline biosynthesis-related genes under salt stress. Moreover, immunoprecipitation-mass spectrometry (IP-MS) assay and firefly luciferase complementation imaging (LCI) assay showed that ZmBSK1 could associate with heat shock protein ZmHSP8 and 14-3-3-like protein ZmGF14-6, and their gene expression levels could be significantly induced by NaCl treatment in different maize tissues. Our findings unravel the new function of ZmBSK1 in salt stress response, which provides the theoretical bases for the improvement of maize salt resistance.

## Introduction

In past decades, plants have been frequently suffered from numerous environmental stimuli during their growth and development, including the biotic stresses resulted from pests and pathogens and the abiotic stresses resulted from salinity, drought, extreme temperature, heavy metals, etc (Atkinson and Urwin, 2012; Suzuki et al., 2014). Among them, salt stress is a major abiotic stress that can induce ionic stress, osmotic stress and secondary stresses such as oxidative stress caused by accumulation of excess reactive oxygen species (ROS; superoxide anion [O_2_^−^], hydroxyl radicals [OH^•^], singlet oxygen [^1^O_2_], and hydrogen peroxide [H_2_O_2_]) in plant cells, thus severely limiting plant growth and crop yield (Yang and Guo, 2018). Many genes, liking protein kinases and transcription factors, have been reported to play important roles in regulating plant tolerance to salt stress by affecting the downstream genes (Yan et al., 2014; Zhang et al., 2014; Yao et al., 2018; Qin et al., 2020).

Brassinosteroid (BR)-signaling kinase (BSK) is a kind of plant-specific protein kinase, which contains a kinase domain in N-terminus and a tetratricopeptide repeat (TPR) domain in C-terminus (Shiu et al., 2004; Kim and Wang, 2010). It belongs to the receptor-like cytoplasmic kinases (RLCKs) subfamily XII (Li et al., 2019). Until now, 12 BSKs in *Arabidopsis* and five BSKs in rice have been identified (Tang et al., 2008; Wang et al., 2017). Many studies have identified the different functions in *Arabidopsis* and rice. For example, AtBSK1, AtBSK2, AtBSK3, and AtBSK5 have been recognized as BR-responsive proteins in BR signaling pathway in *Arabidopsis* (Tang et al., 2008). AtBSK1 and *Oryza sativa* BSK3 (OsBSK3) can directly interact with and be phosphorylated by *Arabidopsis* BR insensitive 1 (AtBRI1) and rice BRI1 (OsBRI1), then the activated BSKs positively regulate BR signaling (Tang et al., 2008; Zhang et al., 2016). Sreeramulu et al. (2013) reveals that AtBSK3, AtBSK4, AtBSK6, AtBSK7, and AtBSK8 play a partial overlapping role in plant growth. However, only AtBSK3 has been found to be a scaffold protein to function in BR-mediated root growth, shoot growth, and organ separation (Ren et al., 2019a). Moreover, AtBSK1, AtBSK5, or AtBSK8 associates with the immune receptors to play crucial roles in activating the pathogen-associated molecular pattern (PAMP)-triggered immunity (Qi et al., 2011; Shi et al., 2013; Yan et al., 2018; Majhi et al., 2019). In rice, OsBSK1-2, an ortholog of AtBSK1, positively regulates flg22- and chitin-triggered defense responses (Wang et al., 2017).

Increasing evidences suggest that BSKs are also involved in responses of plants to abiotic stresses. For example, salinity, alkali (NaHCO_3_), drought, cold, phytohormones BR, and abscisic acid (ABA) can obviously upregulate the transcript levels of *BSKs* gene in many species including *Arabidopsis*, barley (*Hordeum spontaneum* L.), *Populus tomentosa* Carr., hemp (*Cannabis sativa* L.), potato (*Solanum tuberosum* L.), and kentucky bluegrass (*Poa pratensis* L.) (Li et al., 2012; Chen et al., 2019a,b; Yang et al., 2019; Jiang et al., 2021; Kang et al., 2021). In *Arabidopsis*, loss-of-function mutant *bsk5* exhibits sensitivity to salinity and ABA, and further analysis shows that AtBSK5 is required for the tolerance of plants to salt stress and ABA-mediated drought stress (Li et al., 2012). Recently, there are nine BSKs have been identified in maize (Li et al., 2019). Only *Zea mays* BSK1 (ZmBSK1), an ortholog of AtBSK1, has been characterized to enhance drought tolerance (Liu et al., 2021). However, little is known about the biological function of BSKs in maize, especially under salt stress conditions.

In this study, we report that the gene expression of *ZmBSK1* can be induced by NaCl and overexpression of *ZmBSK1* enhances salt tolerance in maize. Further, we unravel the mechanism of ZmBSK1 in salt tolerance.

## Materials and Methods

### Plant Materials, Growth Conditions, and Salt Stress Treatment

Maize (*Zea mays* L.) inbred line B73 (from Nanjing Agricultural University, China) and tobacco (*Nicotiana benthamiana* L., from Nanjing Agricultural University, China) were used in this study. Maize and tobacco seeds were sown on pots containing soil mixture (soil: vermiculite, 1: 1, v/v) or grown in modified 1/2Hoagland nutrient solution (Coolaber, China) in an artificial climate chamber at a temperature of 28°C, photoperiod 14 h light: 10 h dark and 60% relative humidity (RH), and were watered daily. For tissue-specific expression analysis, roots, stems, and leaves were collected in three-leaf stage of maize, and pollens as well as pistils were collected in flowering stage of maize. For salt stress treatment, 10-day-old maize seedlings grown in nutrient solution were treated with or without 200 mM NaCl for various times, and then the second leaves were harvested and used for further analysis.

### Generation of Transgenic Maize Plants

The full-length coding sequence of *ZmBSK1* was cloned into pCUN-NHF expression vector driven by *ubiquitin* promoter with a 3 × Flag tag at the N terminus. The maize inbred line B73 was used as the plant receptor. The *Agrobacterium*-mediated maize transformation was performed as described by Liu et al. (2015). Positive transformants were selected by 75 mg L^−1^ herbicide Basta (Sangon Biotech, China) and were further confirmed by PCR. The primers are listed in Supplementary Table 1. The homozygous T_3_ lines were obtained for analysis of phenotypes, gene expressions, and physiological indexes.

### RNA Extraction and qRT-PCR Analysis

Total RNA were isolated from different maize tissues using RNAiso Plus Kit (Takara, China) following the manufacturer’s protocol, and the cDNA was synthesized using M5 Super plus qPCR RT Kit (Mei5bio, China). Quantitative RT-PCR was performed on a CFX96 Touch System (Bio-Rad, United States) using 2 × M5 HiPer Realtime PCR Super Mix (Mei5bio, China) according to the manufacturer’s protocol. The gene expression levels were calculated by the 2^-ΔΔCT^ method and were normalized against *ZmActin2* gene. The specific primers used for qRT-PCR are listed in Supplementary Table 1.

### Western Blot Analysis

Total proteins were extracted from maize leaves as described previously (Ma et al., 2012), and their content was quantified using Bradford Protein Assay Kit (Beyotime, China) according to the manufacturer’s protocol. Extracted proteins were separated by 12% SDS-PAGE and were transferred to a polyvinylidene fluoride (PVDF) membrane (Merck Millipore, United States). The membrane was blocked in PBST buffer containing 5% [w/v] skimmed milk powder for 2 h at 25°C, and was then incubated with primary antibody including anti-Flag antibody (1:5,000, Abmart) or anti-actin antibody (1:5,000, Biodragon, China). The secondary antibody, horseradish peroxidase (HRP)-conjugated anti-mouse antibody (Abmart), was used at 1:5,000 dilution. Chemifluorescent signal generated by BeyoECL plus (Beyotime, China) western blotting detection reagents was captured by a camera (Tanon 5200 Multi, China).

### Phenotype and Oxidative Damage Analysis

For the root phenotype, maize seeds were spread on the paper containing 200 mM NaCl solution and kept for 4 days, after which the root length were measured. For the growth phenotype, 10-day-old maize seedlings were treated with 200 mM NaCl for 14 days. After recovery by rewatering for 5 days, the survival rate was calculated. For oxidative damage analysis, 10-day-old maize seedlings were treated with 200 mM NaCl for 2 days, the malondialdehyde (MDA) content and the percentage of electrolyte leakage were measured as described by Zhu et al. (2016).

### Detection of H_2_O_2_ and O_2_^−^

About 10-day-old maize seedlings were treated with 200 mM NaCl for 2 days, and the second leaves were harvested for the subsequent analysis. For H_2_O_2_ staining and O_2_^−^ staining, the leaves were incubated in 1 mg ml^−1^ 3,3′-diaminobenzidine (DAB) solution (pH 3.8, Coolaber, China) and 0.5 mg ml^−1^ nitroblue tetrazolium chloride (NBT) solution (Solarbio, China) for 8 h in dark at room temperature, respectively. After staining, the leaves were boiled in 95% ethanol for 10 min to decolorize, and then photographed. For quantification, the contents of H_2_O_2_ and O_2_^−^ were determined using H_2_O_2_ Detection Kit (Leagene, China) and Micro Superoxide Anion Assay Kit (Solarbio, China) according to the manufacturer’s protocol, respectively.

### Measurement of Antioxidant Defense Enzyme Activity and Proline Content

The harvest maize leaves were homogenized in extraction buffer containing 50 mM potassium phosphate (pH 7.0), 1 mM EDTA and 1% (w/v) polyvinylpyrrolidone 40. The homogenates were centrifuged at 12,000 × *g* for 30 min at 4°C, and the supernatants were immediately used for the subsequent antioxidant defense enzyme assays. Total activities of antioxidant defense enzymes were measured as described previously (Zhang et al., 2010). The proline content was determined using Proline Assay Kit (Leagene, China) according to the manufacturer’s instructions.

### Immunoprecipitation-Mass Spectrometry

About 10-day-old OE-*ZmBSK1* transgenic lines were treated with 200 mM NaCl for 0 or 10 h, and then total proteins from the leaves were extracted using lysis buffer [10 mM Tris–HCl, pH 7.5, 150 mM NaCl, 5 mM EDTA, 1% (v/v) Triton X-100, 0.1% (w/v) SDS, 0.5 mM DTT, and protease inhibitor cocktail]. Protein extracts were immunoprecipitated with anti-Flag antibody (1:300, Abmart) bound to protein A/G agarose beads in PBS buffer containing protease inhibitor cocktail for 8 h at 4°C. The beads were washed for three times with PBS buffer and were boiled in SDS loading buffer (Beyotime, China). After centrifugation, the supernatants were processed according to a previous publication (Wang et al., 2015), and were analyzed using EASY-nLC 1200 ultra-high performance liquid system (Thermo Scientific, United States) and Q Exactive HF mass spectrometry system (Thermo Scientific, United States). For protein identification, the MS data were searched against maize database in UniProtKB using the Paragon algorithm in ProteinPilot ™ Software (v5.0.2, SCIEX). For the identified proteins, selected certain filtering criteria and peptides with an unused score > 1.3 were considered as credible peptides, and proteins containing at least one unique peptide were retained.

### Firefly Luciferase Complementation Imaging Assay

The full-length coding sequence of *ZmBSK1* was cloned into vector pCAMBIA1300-nLUC to express ZmBSK1-nLUC fusion protein. The full-length coding sequences of *ZmHSP8* and *ZmGF14-6* were cloned into vector pCAMBIA1300-cLUC to express cLUC-ZmHSP8 and cLUC-ZmGF14-6 fusion proteins, respectively. The empty vectors were used as negative control. *Agrobacterium tumefaciens* strain GV3101 containing each construct was transiently transfected into 4-week-old tobacco leaves. After 3 days of infiltration, 1 mM D-luciferin (PerkinElmer, United States) was sprayed onto the leaves and kept for 20 min in dark, and then the luminescence was captured by a low-light cooled charge coupled device camera (Tanon 5200 Multi, China).

### Statistical Analysis

Statistical analysis was performed using the software SPSS (v16.0).^1^ One-way or two-way ANOVA corrected with Duncan’s multiple range test was used to determine statistical significance. Differences were considered significant at *p* < 0.05.

### Accession Numbers

Sequence data from this article can be found in MaizeGDB database under the following accession numbers: *ZmBSK1*, Zm00001d048345; *ZmActin2*, Zm00001d013873; *ZmcAPX*, Zm00001d007234; *ZmCAT1*, Zm00001d014818; *ZmCSD5*, Zm00001d022505; *ZmMSD2*, Zm00001d009990; *ZmP5CS1*, Zm00001d012391; *ZmP5CS2*, Zm00001d010056; *ZmHSP8*, Zm00001d031325; and *ZmGF14-6*, Zm00001d003401.

## Results

### Expression Patterns of *ZmBSK1*

**Figure 1:**
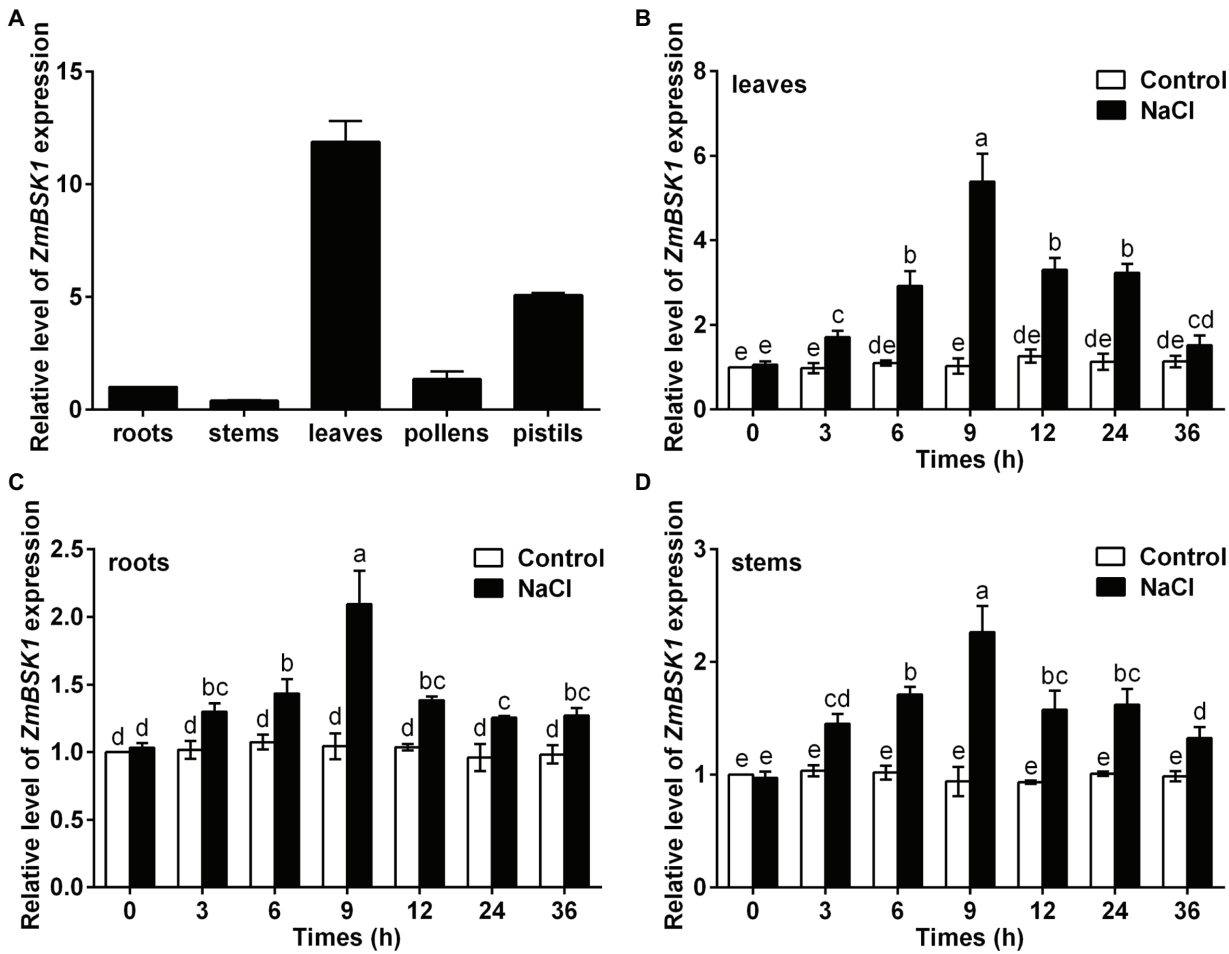
Expression patterns of *ZmBSK1* gene in maize. **(A)** qRT-PCR analysis of the expression levels of *ZmBSK1* in different maize tissues (roots, stems, leaves, pollens, and pistils). **(B-D)** The expression levels of *ZmBSK1* in maize leaves **(B)**, roots **(C)**, and stems **(D)** exposed to salt stress. About 10-day-old wild type seedlings were treated with 200 mM NaCl for indicated times, and the expression was measured by qRT-PCR. *ZmActin2* served as an internal control. Error bars represent ±SD (*n* = 3). Different letters indicate significant differences at *p* < 0.05 according to two-way ANOVA (Duncan’s multiple range test).

To determine the tissue-specific expression level of *ZmBSK1*, qRT-PCR was used to detect the transcript levels of *ZmBSK1* in different organ tissues of maize, including roots, stems, leaves, pollens, and pistils. The results showed that the expression level of *ZmBSK1* was higher in the leaves and pistils, and was lower in the roots and stems (Figure 1A), implying that ZmBSK1 may function in the process of ear development. To explore the effect of salt stress on the expression level of *ZmBSK1* gene, qRT-PCR was conducted after maize plants were treated with or without 200 mM NaCl for various times. We found that the expression level of *ZmBSK1* was gradually induced to a maximum value (5-fold in leaves, 2-fold in roots, and 2.3-fold in stems) within 9 h and then declined after treatment with 200 mM NaCl (Figures 1B–D). The results suggest that ZmBSK1 may play a positive role in plants response to salt stress.

### Overexpression of *ZmBSK1* Enhances Salt Tolerance in Maize

**Figure 2:**
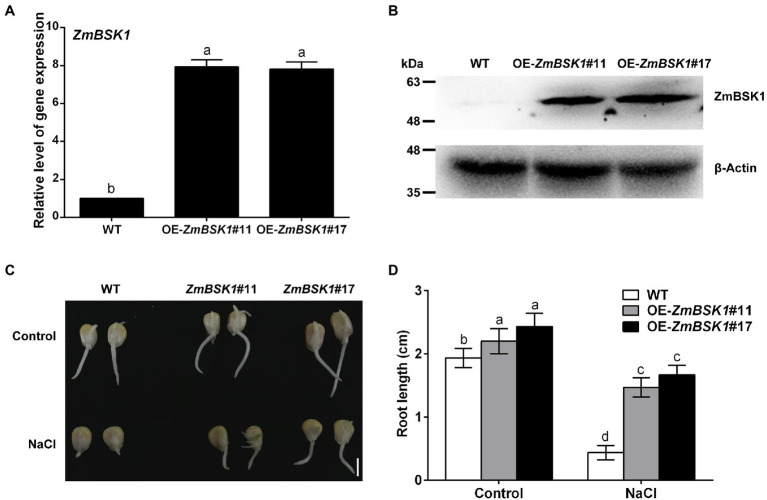
Effect of ZmBSK1 on root growth of maize seeds under salt stress. **(A)** The expression levels of *ZmBSK1* in OE-*ZmBSK1* and wild type (WT) plants. The expression of *ZmBSK1* was measured by qRT-PCR, and *ZmActin2* served as an internal control. **(B)** The protein levels of ZmBSK1 in OE-*ZmBSK1* and WT plants. Total proteins extracted from the leaves were used for immunoblotting with anti-Flag antibody. β-actin was used as a loading control. **(C)** The root growth phenotypes of OE-*ZmBSK1* and WT maize seeds under salt stress. The maize seeds were spread on paper containing 200 mM NaCl for 4 days during germination. Scale bar = 1 cm. **(D)** Statistical analysis of root length in **(C)**. Error bars in **(A,D)** represent ±SD (*n* = 3). Different letters indicate significant differences at *p* < 0.05 according to one-way or two-way ANOVA (Duncan’s multiple range test).

**Figure 3:**
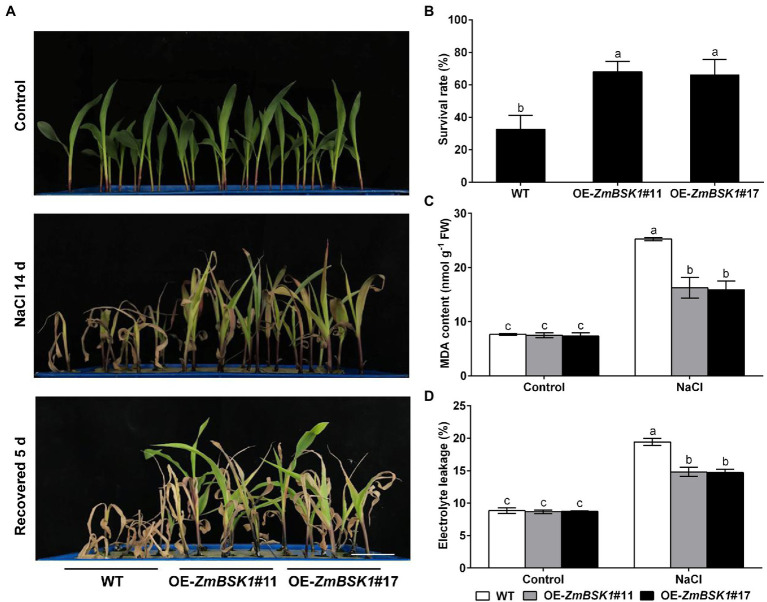
Overexpression of *ZmBSK1* improves salt tolerance in maize. **(A)** The phenotypes of OE-*ZmBSK1* and WT maize plants under salt stress. About 10-day-old maize seedlings were treated with 200 mM NaCl for 14 days, and then recovered for 5 days. Scale bar = 5 cm. **(B)** Survival rate (%) of maize plants in **(A)**. At least 45 seedlings of each line per replicate were used for survival rate analysis. The malondialdehyde (MDA) content **(C)** and the percentage of electrolyte leakage **(D)** in leaves of OE-*ZmBSK1* and WT maize plants under salt stress. About 10-day-old maize seedlings were treated with 200 mM NaCl for 2 days, and then the physiological indexes as indicated were measured. Error bars in **(B–D)** represent ±SD (*n* = 3). Different letters indicate significant differences at *p* < 0.05 according to one-way or two-way ANOVA (Duncan’s multiple range test).

To further investigate the biological function of ZmBSK1 in maize under salt stress, two independent *ZmBSK1*-overexpressing lines (OE-*ZmBSK1*#11 and OE-*ZmBSK1*#17) were generated, then the expressions of ZmBSK1 at transcript and protein levels were confirmed by qRT-PCR and immunobloting assays, respectively (Figures 2A,B). Wild type (WT) and OE-*ZmBSK1* transgenic maize seeds were soaked in 200 mM NaCl solution at the germination stage, and then the root length was measured. As shown in Figures 2C,D, both two OE-*ZmBSK1* maize seeds exhibited slightly longer root length than WT under normal conditions. However, these two transgenic maize seeds developed obviously longer roots than WT when exposed to salt treatment. Subsequently, 10-day-old WT and OE-*ZmBSK1* transgenic seedlings were treated with or without 200 mM NaCl. Under normal conditions, there was no significant difference in the growth phenotypes between WT and transgenic lines (Figure 3A). Under salt stress, OE-*ZmBSK1* lines presented less wilting and chlorosis than WT (Figure 3A). After recovery by rewatering, all transgenic lines had higher survival rates compared with WT plants (Figure 3B). These results indicate that ZmBSK1 positively regulates salt stress tolerance in maize.

### Overexpression of *ZmBSK1* Alleviates Oxidative Damage Caused by Salt Stress in Maize

The MDA content and the percentage of electrolyte leakage are two major indicators of oxidative damage in plants (Jiang and Zhang, 2002; Zhang et al., 2011). Thus, these two physiological indicators were also detected. As shown in Figures 3C,D, without NaCl treatment, no significant difference was observed in the MDA content and the percentage of electrolyte leakage between WT plants and transgenic lines. Salt stress caused marked increases in the MDA content and the percentage of electrolyte leakage in WT plants compared with control conditions, which were further alleviated in OE-*ZmBSK1* transgenic lines. These results suggest that overexpression of *ZmBSK1* can largely protect maize plants from oxidative damage under salt stress.

### ZmBSK1 Reduces the Accumulation of ROS by Enhancing Antioxidant Defense System Under Salt Stress in Maize

**Figure 4:**
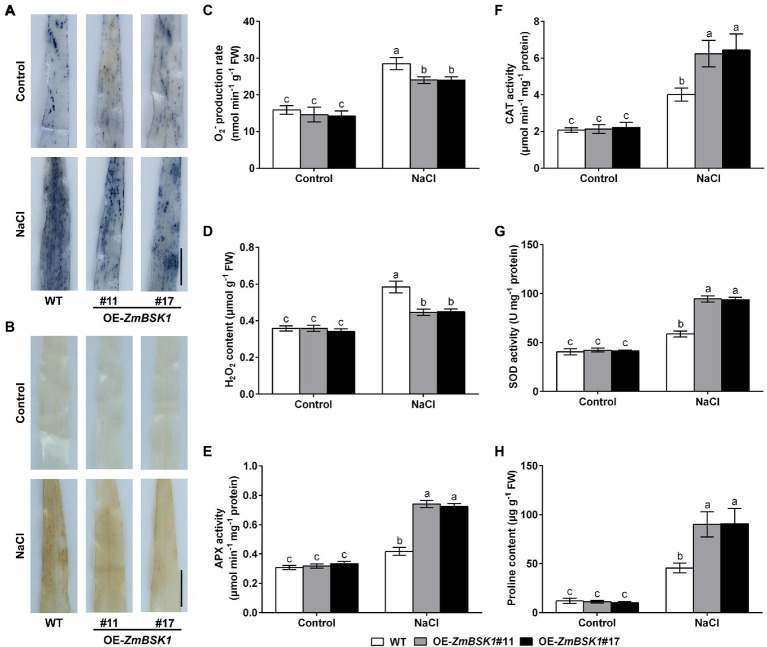
Effect of *ZmBSK1* on reactive oxygen species (ROS) levels, antioxidant defense enzymes activity and proline content under salt stress. Nitroblue tetrazolium (NBT) staining of O_2_^−^ **(A)** and 3,3′-diaminobenzidine (DAB) staining of H_2_O_2_ **(B)** in leaves of OE-*ZmBSK1* and WT maize plants under salt stress. Scale bar = 1 cm. Statistical analysis of O_2_^−^ production rates **(C)** and H_2_O_2_ content **(D)** in leaves of OE-*ZmBSK1* and WT maize plants under salt stress. Ascorbate peroxidase (APX) activity **(E)**, catalase (CAT) activity **(F)**, superoxide dismutase (SOD) activity **(G)**, and proline content **(H)** in leaves of OE-*ZmBSK1* and WT maize plants under salt stress. About 10-day-old maize seedlings in **(A–H)** were treated with 200 mM NaCl for 2 days, and then the above physiological indexes as indicated were measured. Error bars in **(C–H)** represent ±SD (*n* = 3). Different letters indicate significant differences at *p* < 0.05 according to two-way ANOVA (Duncan’s multiple range test).

Salinity-induced ROS accumulation is responsible for oxidative damage (Foyer, 2018). The NBT and DAB staining were used to determine the levels of O_2_^−^ and H_2_O_2_ in maize leaves. As shown in Figures 4A,B, the accumulation of O_2_^−^ and H_2_O_2_ in OE-*ZmBSK1* transgenic lines was obviously lower than that in WT plants exposed to salt stress. In agreement with the results of staining, the O_2_^−^ production rate and the H_2_O_2_ content were lower in transgenic lines than that in WT plants under salt stress (Figures 4C,D). Under normal conditions, there was no obvious difference in the accumulation and the content of ROS in all lines (Figures 4A–D). These results show that overexpression of *ZmBSK1* reduces ROS level in plants in response to salt stress.

Antioxidant defense system is required for scavenging excess ROS produced by various abiotic stresses (Julkowska and Testerink, 2015; You and Chan, 2015; Hasanuzzaman et al., 2021). Accordingly, to explore the effect of ZmBSK1 on the antioxidant defense enzymes in maize in response to salt stress, the activities of three key enzymes ascorbate peroxidase (APX), catalase (CAT), and superoxide dismutase (SOD) were examined. Under normal conditions, both WT plants and OE-*ZmBSK1* transgenic lines had the similar activities of APX, CAT, and SOD. Under salt stress, however, the activities of these three enzymes in transgenic lines increased more than those in WT (Figures 4E–G). In addition to the antioxidant defense enzymes, the content of the non-enzymatic antioxidants such as proline was also measured in maize. Consistent with the results of enzymes activities, the proline content was much higher in all transgenic lines than in WT plants when exposed to salt treatment (Figure 4H). Taken together, these results demonstrate that ZmBSK1 enhances the tolerance of maize plants to salt stress by inducing the activities of antioxidant defense enzymes and the accumulation of proline and alleviating ROS level.

### ZmBSK1 Modulates the Expressions of Stress-Related Genes Under Salt Stress

**Figure 5:**
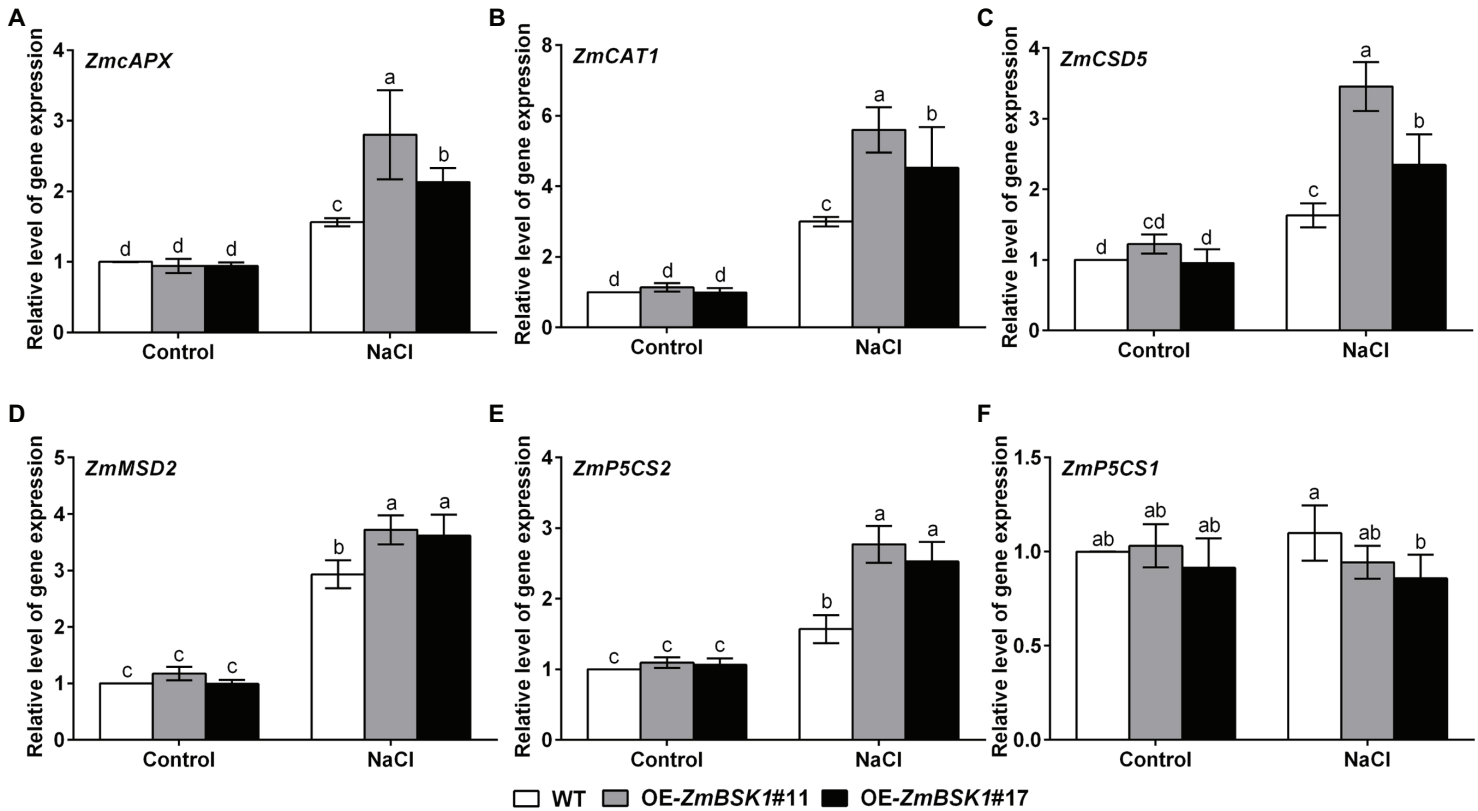
ZmBSK1 positively regulates the expression of stress-related genes in maize under salt stress. The expression levels of stress-related genes *ZmcAPX* **(A)**, *ZmCAT1* **(B)**, *ZmCSD5* **(C)**, *ZmMSD2* **(D)**, *ZmP5CS2* **(E)**, and *ZmP5CS1* **(F)** in OE-*ZmBSK1* and WT maize plants exposed to salt stress. About 10-day-old maize seedlings were treated with 200 mM NaCl for 6 h, and the expressions were measured by qRT-PCR. *ZmActin2* served as an internal control. Error bars in **(A–F)** represent ±SD (*n* = 3). Different letters indicate significant differences at *p* < 0.05 according to two-way ANOVA (Duncan’s multiple range test).

To study the mechanism by which ZmBSK1 functioned in plant tolerance to salt stress, qRT-PCR was used to analyze the expression levels of several selected stress-related genes, including ROS-scavenging genes *ZmcAPX* (cytosolic APX), *ZmCAT1*, *ZmCSD5* (Cu/Zn-SOD), and *ZmMSD2* (Fe/Mn-SOD) as well as proline biosynthesis-related genes *ZmP5CS1* and *ZmP5CS2* (Wang et al., 2014). Under normal conditions, the expression levels of all genes were similar between WT and OE-*ZmBSK1* transgenic lines (Figure 5). After salt treatment, compared with WT plants, *ZmcAPX*, *ZmCAT1*, *ZmCSD5*, *ZmMSD2*, and *ZmP5CS2* genes displayed increased expression levels in the transgenic lines (Figures 5A–E). Nevertheless, among these genes, only *ZmP5CS1* gene could not be induced in all lines (Figure 5F). This might be due to the fact that the expression of *ZmP5CS1* is induced at a slower rate than that of *ZmP5CS2* in the early stage of salt stress. In general, these data reveal that ZmBSK1 improves salt tolerance by modulating the expression levels of stress-related genes.

### ZmBSK1 Interacts With ZmHSP8 and ZmGF14-6

**Figure 6:**
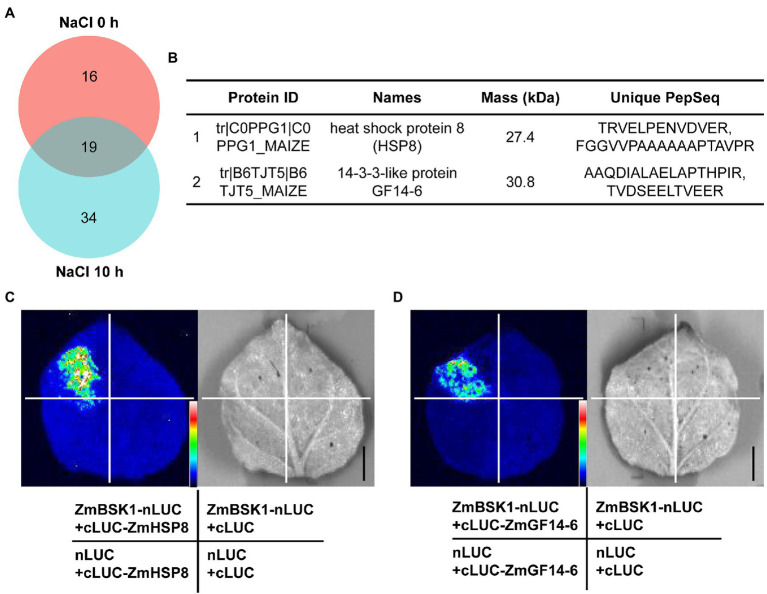
ZmBSK1 interacts with its target proteins ZmHSP8 and ZmGF14-6. **(A)** Venn diagram shows the number of ZmBSK1-interacting proteins in OE-*ZmBSK1* maize plants with or without salt stress. About 10-day-old maize seedlings were treated with 200 mM NaCl for 0 or 10 h. Total proteins extracted from leaves were immunoprecipitated by anti-Flag antibody and the interacting proteins of ZmBSK1 were identified by immunoprecipitation-mass spectrometry (IP-MS). **(B)** The candidate target proteins of ZmBSK1 upon mass spectrometry analysis only after salt treatment. Firefly luciferase complementation imaging (LCI) assays confirm the interactions of ZmBSK1 with ZmHSP8 **(C)** and ZmGF14-6 **(D)**. The combinations of *ZmBSK1-nLUC* and *cLUC-ZmHSP8*, *ZmBSK1-nLUC* and *cLUC-ZmGF14-6* were co-transformed into tobacco leaves. The combinations of *ZmBSK1-nLUC* and *cLUC*, *nLUC* and *cLUC-ZmHSP8*, *nLUC* and *cLUC-ZmGF14-6*, *nLUC* and *cLUC* were used as negative controls. nLUC and cLUC represent the N-terminal and C-terminal fragments of firefly luciferase, respectively. Images were collected from the detached leaves after infiltration for 3 days. Scale bar = 1 cm.

Previous studies have demonstrated that BSKs can directly interact with several kinases and phosphatases, such as BRI1, BRI1-suppressor 1 (BSU1), ZmCCaMK, FLAGELLIN SENSING2 (FLS2), MEK Kinase5 (MAPKKK5), and RECEPTOR-LIKE KINASE 902 (RLK902) (Tang et al., 2008; Kim et al., 2009; Shi et al., 2013; Yan et al., 2018; Zhao et al., 2019; Liu et al., 2021). These findings suggest that BSKs require some different downstream components to regulate different development processes or stress responses. To further understand how ZmBSK1 functions in salt tolerance, immunoprecipitation-mass spectrometry (IP-MS) assay was carried out to identify the interacting proteins of ZmBSK1 in OE-*Flag-ZmBSK1* transgenic lines using anti-Flag antibody under salt stress. Total 69 proteins were detected in this study (Figure 6A; Supplementary Table 2). Among these proteins, heat shock protein ZmHSP8 and 14-3-3-like protein ZmGF14-6 were chose to further confirm their interactions with ZmBSK1 using luciferase complementation imaging (LCI) assay (Figure 6B). ZmBSK1 was fused with the N-terminal fragment of luciferase (ZmBSK1-nLUC), meanwhile, ZmHSP8 and ZmGF14-6 were independently fused with the C-terminal fragment of luciferase (cLUC-ZmHSP8 and cLUC-ZmGF14-6). A strong luminescence signal was observed in tobacco leaves injected with ZmBSK1-nLUC and cLUC-ZmHSP8 (Figure 6C) as well as ZmBSK1-nLUC and cLUC-ZmGF14-6 (Figure 6D). These observations indicate that ZmBSK1 interacts with ZmHSP8 and ZmGF14-6, respectively.

### Expression Analysis of *ZmHSP8* and *ZmGF14-6* Under Salt Stress

**Figure 7:**
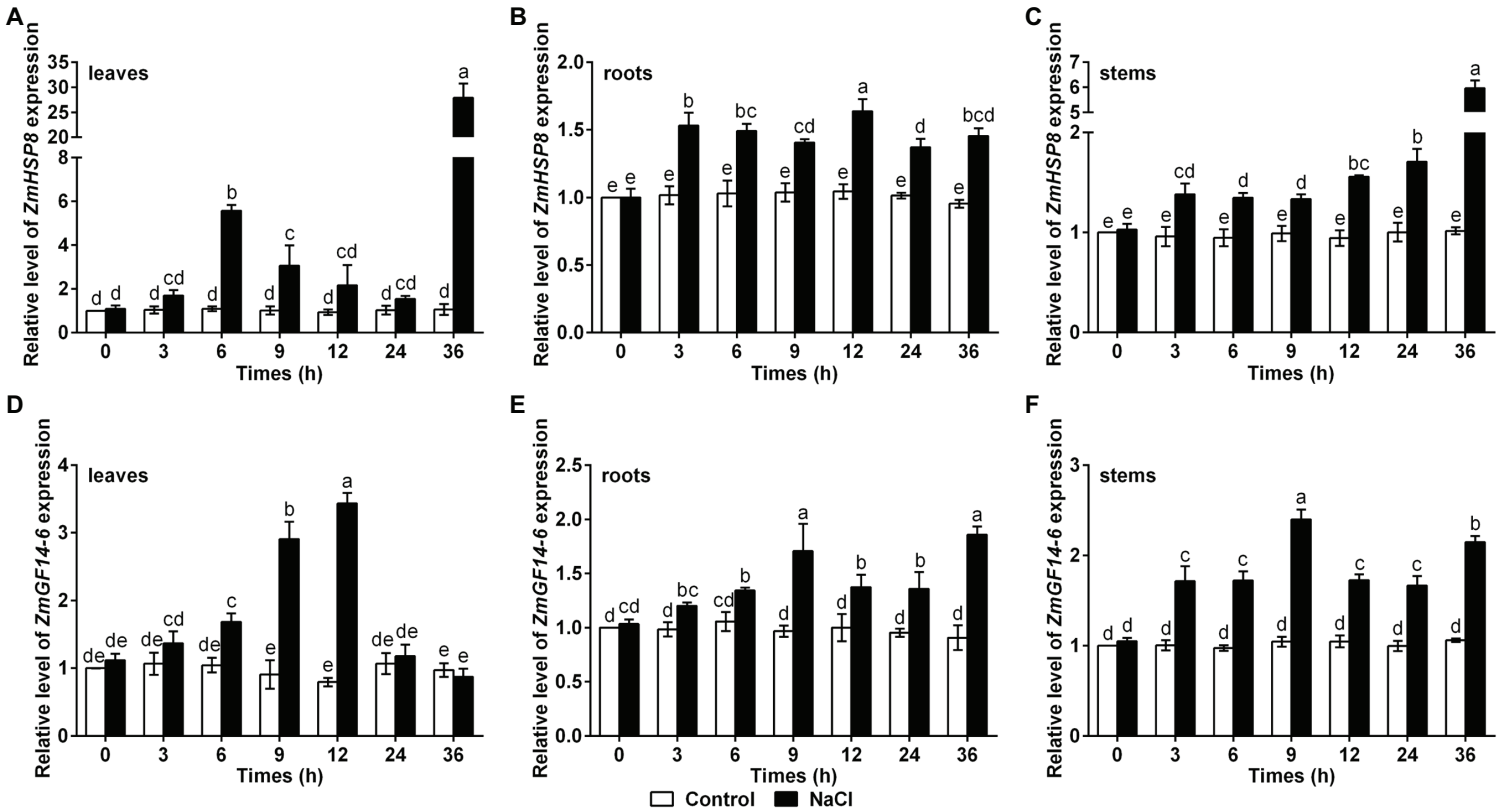
Gene expression patterns of *ZmHSP8* and *ZmGF14-6* under salt stress. **(A–C)** The expression levels of *ZmHSP8* in maize leaves **(A)**, roots **(B)**, and stems **(C)** exposed to salt stress. **(D–F)** The expression levels of *ZmGF14-6* in maize leaves **(D)**, roots **(E)**, and stems **(F)** exposed to salt stress. About 10-day-old wild type seedlings were treated with 200 mM NaCl for indicated times, and the expression was measured by qRT-PCR. *ZmActin2* served as an internal control. Error bars represent ±SD (*n* = 3). Different letters indicate significant differences at *p* < 0.05 according to two-way ANOVA (Duncan’s multiple range test).

Since ZmHSP8 and ZmGF14-6 could directly interact with ZmBSK1, we wondered whether they functioned in salt response in maize. To test this, qRT-PCR was used to detect the transcript levels of *ZmHSP8* and *ZmGF14-6* genes in maize with or without NaCl treatment. As shown in Figure 7, under salt stress, the expression of *ZmHSP8* gene was rapidly induced to reach the first peak value at 6 h and the second peak value at 36 h in leaves and was gradually induced to the peak value at 36 h in stems. Moreover, under salt stress, the expression of *ZmGF14-6* gene gradually increased to its highest level within 12 h in leaves and 9 h in stems, whereas both *ZmHSP8* and *ZmGF14-6* were slightly upregulated in roots. These findings imply that ZmHSP8 and ZmGF14-6 may play a role in improving plant tolerance to salt stress.

## Discussion

Brassinosteroid (BR)-signaling kinases are first identified in *Arabidopsis* as a brassinosteroid-signaling kinase that is found to play a role in BR signaling pathway (Tang et al., 2008). Up to now, BSKs have been well characterized to play vital roles in many other biological processes such as plant growth and development as well as plant immunity (Sreeramulu et al., 2013; Yan et al., 2018). In addition, an increasing number of studies have demonstrated that BSKs are also involved in regulating plant responses to abiotic stresses (Li et al., 2012; Chen et al., 2019a; Yang et al., 2019; Liu et al., 2021). There are 9 BSKs have been found in maize (Li et al., 2019); however, the roles of ZmBSKs in response to abiotic stresses remain largely unknown.

In the present study, we revealed that ZmBSK1 acted as a positive regulator in plant salt tolerance, in agreement with AtBSK5 in a previous study (Li et al., 2012), based on the evidence that OE-*ZmBSK1* transgenic lines displayed a better growth performance and a higher survival rate under salt stress (Figures 2, 3A,B). Moreover, changes of diverse physiological indicators appear to reflect the mechanisms by which transgenic lines cope with environmental stimuli. As previously reported, the expression of *BSKs* genes can be induced by different abiotic stresses (Yang et al., 2019; Jiang et al., 2021; Kang et al., 2021). And interestingly, numerous salinity stress-related *cis*-elements such as ARE were detected in the promoter region of *ZmBSK1* (Supplementary Table 3), which might be responsible for the significant increase in *ZmBSK1* gene expression under salt stress conditions (Figures 1B–D). The antioxidant defense system can be activated to mitigate oxidative damage caused by ROS under stress conditions (Sharma et al., 2012; Xiang et al., 2021; Yan et al., 2022). Our results showed that the MDA content and the percentage of electrolyte leakage in OE-*ZmBSK1* transgenic lines were lower than those in WT plants (Figures 3C,D). Oxidative damage is tightly linked to the ROS accumulation (Gong et al., 2005; Li et al., 2008; Yang and Guo, 2018). Similarly, lower O_2_^−^ and H_2_O_2_ levels were also observed in transgenic lines (Figures 4A–D), indicating that transgenic lines had lower ROS accumulation and oxidative damage degree under salt treatment. To protect plants from oxidative damage, the antioxidant defense system would be rapidly induced to scavenge excess ROS (Das and Roychoudhury, 2014; Moradbeygi et al., 2020). As expected, transgenic lines exhibited higher APX, CAT, and SOD activities and proline content (Figures 4E–H), contributing to maintain low ROS levels and the balance of osmotic pressure. The expressions of stress-responsive genes are crucial for enhancing plant tolerance to various stresses (Chini et al., 2004; Ren et al., 2019b). Indeed, a higher expressions of ROS-scavenging enzyme genes *ZmcAPX*, *ZmCAT1*, *ZmCSD5*, and *ZmMSD2* were observed in transgenic lines (Figures 5A–D), which were consistent with the differences in the activities of ROS-scavenging enzymes between WT and transgenic lines under NaCl treatment. Furthermore, *P5CS1/2* encodes a rate-limiting enzyme in the biosynthesis of proline (Wang et al., 2014), which can be upregulated under salt stress in maize (Wang et al., 2013). Likewise, in current study, the expression of *ZmP5CS2* could be further induced in transgenic lines than that in WT plants under salt stress (Figure 5E). However, *ZmP5CS1* showed the similar expressions in both transgenic lines and WT plants with or without NaCl treatment (Figure 5F). Due to the differences in NaCl processing time, one possible explanation is that *ZmP5CS1* has not been induced during the initial stage of salt stress.

As a receptor-like protein kinase, BSKs are investigated to function in diverse biological processes by interacting with and phosphorylating its target proteins such as BSU1, MAPKKK5, and ZmCCaMK (Tang et al., 2008; Yan et al., 2018; Liu et al., 2021), which means that there are some unknown interactors of ZmBSK1 under salt stress conditions. Our IP-MS results revealed two novel interacting proteins of ZmBSK1 during NaCl treatment, ZmHSP8 and ZmGF14-6 (Figure 6). The molecular mechanisms underlying the functions of HSPs in biotic stress signaling, drought stress signaling, hormone signaling, and development have been extensively studied in many species (Jacob et al., 2017). Recently, transcriptomic analysis showed that *ZmHSP8* was involved in response to drought and heat stresses in maize (Qian et al., 2019; Blein-Nicolas et al., 2020), whereas we found that NaCl treatment can obviously upregulate *ZmHSP8* gene expression (Figures 7A–C), implying that *ZmHSP8* may play a role in salt stress response. In animals, it is known that the phosphorylation of HSPs by stress kinase is one of the most important post-translational modifications, which functions in enhancing chaperone activities and its affinity for unfolded proteins during stress response (Späth et al., 2015). However, there are few reports on the roles of HSPs phosphorylation in plants. Recently, Zhao et al. (2021) finds that maize sHSP17.4 can interact with and be phosphorylated by ZmCDPK7 in regulating heat stress response. Furthermore, due to the lack of catalytic activity, 14-3-3 proteins usually function *via* physical interactions. Previous studies have reported that 14-3-3 proteins interact with various target proteins to regulate diverse biological processes, including metabolism, transcription, protein trafficking, and stress responses (Roberts, 2003; Zhou et al., 2014; Zhang et al., 2018). Additionally, the phosphorylation of 14-3-3 proteins also plays an important role in their functions. In *Arabidopsis*, a plasma membrane-localized kinase CRPK1 can phosphorylate 14-3-3 proteins, which followed by translocating from the cytoplasm into the nucleus to regulate the stability of CBFs in cold response (Liu et al., 2017). In maize, the gene expression of *ZmGF14-6*, encoding a 14-3-3-like protein, could be upregulated by salt stress (Campo et al., 2012), which was consistent with our findings (Figures 7D–F). Because ZmBSK1 is also a plasma membrane-anchored kinase, we wonder if it can recruit and phosphorylate ZmGF14-6 to further modulate salt tolerance. Here, our data provided the possibility of ZmBSK1 and ZmHSP8 or ZmGF14-6 in improving plant tolerance to salt stress through interacting with each other.

Based on these results, we provide a working model of ZmBSK1 in salt response. Salt stress rapidly induces the expression of *ZmBSK1*, thus upregulating the expressions of salt stress-responsive genes to enhance antioxidant defense enzyme activities and promote proline synthesis, improving the salt tolerance. In this process, the two interacting proteins of ZmBSK1, ZmHSP8, and ZmGF14-6, are identified and might function in salt response. Future work is needed to clarify the molecular mechanisms by which ZmBSK1 and its two interacting proteins positively regulate plant salt tolerance.

## Data Availability Statement

The original contributions presented in the study are included in the article/Supplementary Material, further inquiries can be directed to the corresponding authors.

## Author Contributions

JY, YC, and LL conceived the project and designed the experiments. LL performed most of the experiments, analyzed the data, and wrote the manuscript. YS performed the phenotype analysis. PD provided the resources of transgenic maize. YC, QM, and XW helped to analyze the data. JY and YC revised the manuscript. All authors contributed to the article and approved the submitted version.

## Funding

This work was supported by the Postdoctoral Science Foundation of Jiangsu Province (2021K406C); the China Agriculture Research System (CARS-02); the Jiangsu Agriculture Science and Technology Innovation Fund [CX(21)3115]; and the earmarked fund for Jiangsu Agricultural Industry Technology System [JATS(2020)385].

## Conflict of Interest

The authors declare that the research was conducted in the absence of any commercial or financial relationships that could be construed as a potential conflict of interest.

## Publisher’s Note

All claims expressed in this article are solely those of the authors and do not necessarily represent those of their affiliated organizations, or those of the publisher, the editors and the reviewers. Any product that may be evaluated in this article, or claim that may be made by its manufacturer, is not guaranteed or endorsed by the publisher.

## Footnotes

^1^ https://www.ibm.com/products/spss-statistics
