# Supplementary material for: Overexpression of a Zea mays Brassinosteroid-Signaling Kinase Gene ZmBSK1 Confers Salt Stress Tolerance in Maize
Source: Front Plant Sci. 2022 May 6;13:894710. doi: 10.3389/fpls.2022.894710 (PMC9121125; doi:10.3389/fpls.2022.894710)
Supplement: Supplementary file 1 [file Table_1.DOCX]

Supplementary Material

## Supplementary Table 1. Primers used in this study.

| **Description** | **Primer name** |  | **Primer Sequences (5’-3’)** |
| --- | --- | --- | --- |
| For interaction analysis | pCAMBIA1300-nLUC-ZmBSK1 | F | ggatccATGGGCTGCTGCGGCTCC |
|  |  | R | acgcgtAAGGTTTCGTGTTCTTCTGCC |
|  | pCAMBIA1300-cLUC-ZmHSP8 | F | ggtaccATGTGGCACGCCGCGGCGG |
|  |  | R | ggatccCTGCACCTGGATGTTGACGA |
|  | pCAMBIA1300-cLUC-ZmGF14-6 | F | ggtaccATGGCATCAGCAGAGCTTT |
|  |  | R | ggatccCTGCCCCTCACTCGAGTCCC |
| For construction of the transgenic maize plant | pCUN-NHF-ZmBSK1 | F | aagcttAATGGGCTGCTGCGGCTCC |
|  |  | R | actagtTCAAGGTTTCGTGTTCTTCTGCC |
| For identification of the transgenic maize plant | pCUN-NHF | gF | ATCGAGACAAGCACGGTCAA |
|  |  | gR | AAACCCACGTCATGCCAGTT |
| For qRT-PCR analysis | ZmActin2 | qF | GCCATCCATGATCGGTATGG |
|  |  | qR | GTCGCACTTCATGATGGAGTTG |
|  | ZmBSK1 | qF | ACCTCCATCCCGTGCTCTTG |
|  |  | qR | GGGTGTTGCGGTTGTGGAG |
|  | ZmHSP8 | qF | TTGTTTGCTATGTCACACATGG |
|  |  | qR | AATTCTCTTGCGTTCGATTCTG |
|  | ZmGF14-6 | qF | CTGACCAAGATCTGTGATGG |
|  |  | qR | GTGAGTTGGAGCAAGCTCAGCCA |
|  | ZmcAPX | qF | TGAGCGACCAGGACATTG |
|  |  | qR | GAGGGCTTTGTCACTTGGT |
|  | ZmCAT1 | qF | TGGAGGGCTTTGGTGTCAAT |
|  |  | qR | TAGATCCTTCGTCGCATGGC |
|  | ZmCSD5 | qF | TCCATGATCAGAGAGCACTA |
|  |  | qR | ACCTCCAGTAGTGTCTTCTT |
|  | ZmMSD2 | qF | CAAACCTTAACCTCCCCTTC |
|  |  | qR | ACTCGACTCTCGTAGGTG |
|  | ZmP5CS1 | qF | ACTGCAATGTCCACTTATCC |
|  |  | qR | TAACCTAGACTAGACACAGC |
|  | ZmP5CS2 | qF | GTTCAGTCATTGTTTGTGTAG |
|  |  | qR | ATAAGAAACAAGTCACCAACAA |

The lowercase letters indicate the restriction enzyme sites.
